# The RNASeq-er API—a gateway to systematically updated analysis of public RNA-seq data

**DOI:** 10.1093/bioinformatics/btx143

**Published:** 2017-03-22

**Authors:** Robert Petryszak, Nuno A Fonseca, Anja Füllgrabe, Laura Huerta, Maria Keays, Y Amy Tang, Alvis Brazma

**Affiliations:** Functional Genomics Group, European Molecular Biology Laboratory, European Bioinformatics Institute, EMBL-EBI, Hinxton, UK

## Abstract

**Motivation:**

The exponential growth of publicly available RNA-sequencing (RNA-Seq) data poses an increasing challenge to researchers wishing to discover, analyse and store such data, particularly those based in institutions with limited computational resources. EMBL-EBI is in an ideal position to address these challenges and to allow the scientific community easy access to not just raw, but also processed RNA-Seq data. We present a Web service to access the results of a systematically and continually updated standardized alignment as well as gene and exon expression quantification of all public bulk (and in the near future also single-cell) RNA-Seq runs in 264 species in European Nucleotide Archive, using Representational State Transfer.

**Results:**

The RNASeq-er API (Application Programming Interface) enables ontology-powered search for and retrieval of CRAM, bigwig and bedGraph files, gene and exon expression quantification matrices (Fragments Per Kilobase Of Exon Per Million Fragments Mapped, Transcripts Per Million, raw counts) as well as sample attributes annotated with ontology terms. To date over 270 00 RNA-Seq runs in nearly 10 000 studies (1PB of raw FASTQ data) in 264 species in ENA have been processed and made available via the API.

**Availability and Implementation:**

The RNASeq-er API can be accessed at http://www.ebi.ac.uk/fg/rnaseq/api. The commands used to analyse the data are available in supplementary materials and at https://github.com/nunofonseca/irap/wiki/iRAP-single-library.

**Supplementary information:**

Supplementary data are available at *Bioinformatics* online.

## 1 Introduction

**Fig. 1:**
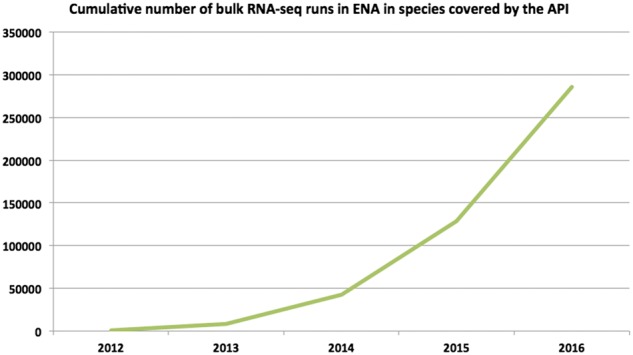
Cumulative number of public bulk RNA-Seq runs in ENA, in species covered by the API

The pattern of rapid growth of RNA-sequencing (RNA-Seq) data, observed in recent years, is set to continue as costs of sequencing experiments decrease and novel technologies and analysis methods reach maturity, e.g. single-cell RNA-Seq (Linnarson *et al.*, 2016). Figure 1 highlights sustained exponential growth in the number of public bulk RNA-Seq runs in European Nucleotide Archive (ENA).

**Fig. 2:**
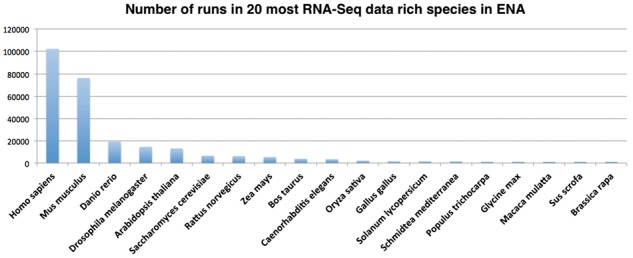
The number of sequencing runs in the top 20 RNA-Seq data-rich species in ENA

A ‘run’ is a unit of biological assay performed on a sequencing machine for a single, de-multiplexed sequencing library preparation. Figure 2 shows the number of runs in the top 20 RNA-Seq data-rich species in ENA.

This sustained growth only exacerbates the challenges facing researchers wishing to discover, analyse and store available RNA-Seq data, particularly those based in institutions with limited computational resources. EMBL-EBI is in an ideal position to address these challenges and to allow the scientific community easy access to not just raw, but also processed RNA-Seq data. We have therefore undertaken the task of on-going standardized alignment and gene and exon expression quantification of all public bulk (and in the near future also single-cell) RNA-Seq data in ENA (Silvester *et al.*, 2014) in 264 species with genome references in Ensembl (Cunningham *et al.*, 2015), Ensembl Genomes (Kersey *et al.*, 2014) and WormBase Parasite (Howe *et al.*, 2016), depositing the results on the public EMBL-EBI FTP server, and making them discoverable via the RNASeq-er API (Application Programming Interface). Our fully automated analysis pipeline processes new RNA-Seq runs as soon as they become public in ENA and makes the results available via the API shortly after. In addition, all RNA-Seq runs in a given species are re-processed when a new genome assembly is released. While the initial processing of the bulk of public RNA-Seq data took around 6 months, the pipeline (utilising 2000 cores in parallel) is capable of processing around 500-1000 sequencing runs per day and thus provides results for any new run in ENA within days of it becoming public. The re-processing for new genome assembly typically takes a week or 2, with the exception of human and mouse (due to the sheer volume of data) and of large genome species (it took over a month to re-process all wheat runs after the new TGACv1 genome reference was released). The RNASeq-er API enables ontology-powered search for and retrieval of CRAM, bigwig and bedGraph files at individual ENA run level, and of gene and exon expression quantification matrices [Fragments Per Kilobase Of Exon Per Million Fragments Mapped (FPKM), Transcripts Per Million (TPM), raw counts] at ENA study level. The API returns data in tab-delimited and JSON formats, and provides additional search filter by the minimum percentage of reads mapped to the genome reference in a given run. The API also provides access to baseline gene expression quantifications, aggregated across all runs in each of over 4000 normal tissue, cell type, developmental stage, sex and strain conditions in 61 species. Please note that it is up to the user of the API to specify the minimum desired percentage of mapped reads—no such filtering is employed by the API a priori. To facilitate discoverability and to allow for interpretation of the analysed data, the API also provides sample attributes per run, including corresponding ontology terms derived from manual curation in ArrayExpress (Kolesnikov *et al.*, 2015) and Expression Atlas (Petryszak *et al.*, 2016). Where manually curated sample annotations are not available, BioSamples database (Faulconbridge *et al.*, 2013) records are used instead. This API has also been incorporated into BioServices Python Package (Cokelaer *et al.*, 2013) and CPAN Perl package (http://search.cpan.org/dist/Bio-EBI-RNAseqAPI/). The analysis pipeline behind the RNASeq-er API offers an important service to researchers performing RNA-Seq experiments that choose to submit their data to ArrayExpress via https://www.ebi.ac.uk/fg/annotare submission tool: the deposited studies are not only described by rich, ontology-annotated experimental metadata; the associated raw data is also analysed for free, and for qualifying studies, is subsequently visualized in Expression Atlas (via private access if pre-publication). This combined metadata-rich deposition, analysis and visualization service aims to make data depositions not only easily discoverable, but also to facilitate understanding and reproducibility of the underlying research results. The results of our analysis can also inform and feed into the submitters’ own downstream analyses well before the paper is ready for submission to a journal.

## 2 Implementation

The analysis of each sequencing run is performed using the iRAP pipeline (Fonseca *et al.*, 2014). First quality-filtered (Petryszak *et al.*, 2014, Supplementary Material) reads are aligned to the latest genome reference via TopHat 2 (Kim *et al.*, 2013). Note that so far we have used STAR (Dobin *et al.*, 2013) for the wheat genome reference, but now that TopHat 2 has been improved to handle large genome references, we plan to use TopHat 2 only for all species. Then the resulting BAM (Li *et al.*, 2009) file is converted to CRAM (Fritz *et al.*, 2011) format; bigWig (https://genome.ucsc.edu/goldenpath/help/bigWig.html) and bedGraph (https://genome.ucsc.edu/goldenpath/help/bedgraph.html) genome track files are also generated. Where groups of technical replicates corresponding to a single biological sample were identified via manual curation in ArrayExpress, the corresponding CRAM, bigWig and bedGraph files are aggregated for each such biological replicate. The expressions (raw counts) of genes and exons defined in the corresponding GTF file (obtained from the same source as the genome reference) are quantified using HTSeq (Anders *et al.*, 2015) and DEXSeq (Anders *et al.*, 2012) respectively. FPKM and TPM are then calculated. The gene lengths are based on the union of exons. Finally, for each gene the median TPM expression and coefficient of variation are calculated across all runs that have the same unique combination of sample attributes, including tissue, cell type, developmental stage, sex and strain.

The full API documentation is available in the Supplementary data. The latest API documentation is also available at http://www.ebi.ac.uk/fg/rnaseq/api/(html) and http://www.ebi.ac.uk/fg/rnaseq/api/doc (pdf).

## Supplementary Material

Supplementary Data
