# Supplementary material for: The RNASeq-er API—a gateway to systematically updated analysis of public RNA-seq data
Source: Bioinformatics. 2017 Mar 22;33(14):2218–20. doi: 10.1093/bioinformatics/btx143 (PMC5870697; doi:10.1093/bioinformatics/btx143)
Supplement: Supplementary Data [file btx143_supp.zip › btx143-suppl_data/RNASeq_API_2016_Application_Note_supplementary_API_Spec.docx]

The RNASeq-er API – version 1.2
1st July 2016

A simple RESTful API to access analysis results of all public
RNA-seq data for 264 species in European Nucleotide Archive.

Robert Petryszak^1,*^, Nuno A. Fonseca^1^, Anja Füllgrabe^1^, Laura Huerta^1^, Maria Keays^1^, Y. Amy Tang^1^, Alvis Brazma^1^

^1^ European Molecular Biology Laboratory, European Bioinformatics Institute, EMBL-EBI, Hinxton, UK
* Contact: [rnaseq@ebi.ac.uk](mailto:rnaseq@ebi.ac.uk)

Contact: [rnaseq@ebi.ac.uk](mailto:rnaseq@ebi.ac.uk)

This document describes the RESTful API that was developed to provide easy access to the results of analysis of public RNA-seq data in [European Nucleotide Archive (ENA)](http://www.ebi.ac.uk/ena). The analysis of each sequencing run was performed by the EMBL-EBI’s [Gene Expression Team](http://www.ebi.ac.uk/about/people/robert-petryszak) using the [iRAP](http://nunofonseca.github.io/irap/) pipeline. Firstly quality-filtered reads were aligned to the latest genome reference from Ensembl via [TopHat 2](https://ccb.jhu.edu/software/tophat/index.shtml) (and [STAR](http://www.ncbi.nlm.nih.gov/pubmed/23104886) for large genomes, e.g. wheat), then the resulting BAM file was converted to [CRAM](http://www.ebi.ac.uk/ena/software/cram-toolkit) format. Finally expression of genes and exons in the corresponding Ensembl GTF file was quantified using [HTSeq](http://www-huber.embl.de/users/anders/HTSeq/doc/overview.html) and [DEXSeq](http://bioconductor.org/packages/release/bioc/html/DEXSeq.html) respectively.

We have extended the iRAP pipeline to analyse public RNA-seq data in the most ’RNA-seq data-rich’ organisms present in ENA. To date, data in 264 organisms have been analysed, including:

[40 in Ensembl](http://www.ebi.ac.uk/fg/rnaseq/api/tsv/0/getOrganisms/ensembl)
[73 in Ensembl Plants](http://www.ebi.ac.uk/fg/rnaseq/api/tsv/0/getOrganisms/plants)
[91 in Ensembl Fungi](http://www.ebi.ac.uk/fg/rnaseq/api/tsv/0/getOrganisms/fungi)
[15 in Ensembl Metazoa](http://www.ebi.ac.uk/fg/rnaseq/api/tsv/0/getOrganisms/metazoa)
 [5 in Ensembl Protists](http://www.ebi.ac.uk/fg/rnaseq/api/tsv/0/getOrganisms/protists)
[38 in WormBase ParaSite](http://www.ebi.ac.uk/fg/rnaseq/api/tsv/0/getOrganisms/wbps)

The pipeline analyses sequencing runs as soon as they become public in ENA, with the results available via the RESTful API shortly after. The annotation of the sequencing metadata to [Experimental Factor Ontology (EFO)](http://www.ebi.ac.uk/efo/) is performed at scale for each new release of EFO, via a tool called [Zooma](http://www.ebi.ac.uk/spot/zooma/). The Zooma knowledgebase from which annotations are derived is based on the manual curation of ENA’s sequencing metadata in [ArrayExpress](http://www.ebi.ac.uk/arrayexpress) and [Expression Atlas](http://www.ebi.ac.uk/gxa), performed by the curators in the [Gene Expression Team](http://www.ebi.ac.uk/about/people/robert-petryszak). If you have any questions, problems using the API or would like us to add to the analysis new organisms of interest please contact the email address above.

This API has also been incorporated into [BioServices Python Package](https://pythonhosted.org/bioservices/references.html#module-bioservices.rnaseq_ebi) and [CPAN Perl Package](http://search.cpan.org/~mkeays/Bio-EBI-RNAseqAPI-1.04/lib/Bio/EBI/RNAseqAPI.pm) .

# Analysis Results Per Run

## Format

| Item | Description |
| --- | --- |
| URL PATTERN | http://www.ebi.ac.uk/fg/rnaseq/api//FORMAT/MAPPING_QUALITY/getRun... |
| FORMAT | tsv or json |
| MAPPING_QUALITY | Minimum percentage of reads mapped to genome reference |
| ORGANISM | See [Ensembl](http://www.ebi.ac.uk/fg/rnaseq/api/tsv/0/getOrganisms/ensembl), [Plants](http://www.ebi.ac.uk/fg/rnaseq/api/tsv/0/getOrganisms/plants), [Fungi](http://www.ebi.ac.uk/fg/rnaseq/api/tsv/0/getOrganisms/fungi), [Metazoa](http://www.ebi.ac.uk/fg/rnaseq/api/tsv/0/getOrganisms/metazoa), [Protists](http://www.ebi.ac.uk/fg/rnaseq/api/tsv/0/getOrganisms/protists) and   [WormBase ParaSite](http://www.ebi.ac.uk/fg/rnaseq/api/tsv/0/getOrganisms/wbps) |
| CONDITION | Check if term exists in EFO, e.g. [cancer](http://www.ebi.ac.uk/efo/search?query=cancer) |

## Example Calls to Retrieve Individual Run Data

| URL |
| --- |
| <http://www.ebi.ac.uk/fg/rnaseq/api/tsv/70/getRunsByOrganism/oryza_longistaminata> |
| [http://www.ebi.ac.uk/fg/rnaseq/api/tsv/70/getRunsByOrganismCondition/homo_sapiens/central nervous system](http://www.ebi.ac.uk/fg/rnaseq/api/tsv/70/getRunsByOrganismCondition/homo_sapiens/central%20nervous%20system) |
| <http://www.ebi.ac.uk/fg/rnaseq/api/tsv/90/getRunsByStudy/SRP033494> |
| <http://www.ebi.ac.uk/fg/rnaseq/api/json/70/getRun/SRR1042759> |

## Returned Fields

| Field | Description |
| --- | --- |
| ASSEMBLY_USED | Genome reference assembly name |
| BIOREP_ID | ENA Run ID or a unique label for technical replicates in RUN_IDS |
| ENA_LAST_UPDATED | Date ENA record for any RUN_IDs was last updated |
| CRAM_LOCATION | FTP location of the CRAM file |
| BEDGRAPH_LOCATION | FTP location of the bedGraph file |
| BIGWIG_LOCATION | FTP location of the BigWig file |
| LAST_PROCESSED_DATE | Date any RUN_IDs were last analysed |
| ORGANISM | Organism of samples in SAMPLE_IDS |
| MAPPING_QUALITY | Percentage of reads mapped to the genome reference |
| REFERENCE_ORGANISM | Genome reference organism |
| RUN_IDS | List of ENA Run ID’s corresponding to BIOREP_ID |
| SAMPLE_ATTRIBUTE_TYPE | Matched sample attribute type |
| SAMPLE_ATTRIBUTE_VALUE | Matched sample attribute value |
| SAMPLE_IDS | BioSamples DB ID’s corresponding to BIOREP_ID |
| STATUS | Processing status in our analysis pipeline |
| STUDY_ID | ENA Study ID |

# Analysis Results Per Study

## Format

## Example Calls to Retrieve Individual Run and Study Data

| URL |
| --- |
| <http://www.ebi.ac.uk/fg/rnaseq/api/tsv/getStudiesByOrganism/arabidopsis_thaliana> |
| <http://www.ebi.ac.uk/fg/rnaseq/api/json/getStudy/SRP033494> |

## Returned Fields

| Field | Description |
| --- | --- |
| ASSEMBLY_USED | Genome reference assembly name |
| GENES_FPKM_COUNTS_FTP_LOCATION | FTP location of gene FPKM counts |
| GENES_TPM_COUNTS_FTP_LOCATION | FTP location of gene TPM counts |
| GENES_RAW_COUNTS_FTP_LOCATION | FTP location of gene RAW counts |
| EXONS_FPKM_COUNTS_FTP_LOCATION | FTP location of exon FPKM counts |
| EXONS_TPM_COUNTS_FTP_LOCATION | FTP location of exon TPM counts |
| EXONS_RAW_COUNTS_FTP_LOCATION | FTP location of exon RAW counts |
| GTF_USED | GTF file used in expression quantification |
| LAST_PROCESSED_DATE | Date the run(s) were last analysed |
| ORGANISM | Organism studied in STUDY_ID |
| REFERENCE_ORGANISM | Genome reference organism |
| SOFTWARE_VERSIONS_FTP_LOCATION | FTP location of pipeline tools info |
| STATUS | Processing status |
| STUDY_ID | ENA Study ID |

# Sample Attributes Per Run

## Format

| Item | Description |
| --- | --- |
| URL PATTERN | http://www.ebi.ac.uk/fg/rnaseq/api/FORMAT/getSampleAttributes... |
| FORMAT | tsv or json |

## Example Calls to Retrieve Individual Run Data

| URL |
| --- |
| <http://www.ebi.ac.uk/fg/rnaseq/api/tsv/getSampleAttributesByRun/SRR805786> |
| <http://www.ebi.ac.uk/fg/rnaseq/api/tsv/getSampleAttributesPerRunByStudy/SRP020492> |
| <http://www.ebi.ac.uk/fg/rnaseq/api/tsv/getSampleAttributesCoverageByStudy/SRP020492> Example Calls to Retrieve Distinct Sample Attributes Across All Runs |
| URL |
| <http://www.ebi.ac.uk/fg/rnaseq/api/tsv/getSampleAttributes> |

##

## Returned Fields

| Field | Description |
| --- | --- |
| EFO_URL | URL of EFO term matching VALUE |
| RUN_ID | ENA Run ID |
| STUDY_ID | ENA Study ID |
| TYPE | Sample Attribute Type |
| VALUE | Sample Attribute Value |
| NUM_OF_RUNS | Number of runs annotated with TYPE/VALUE |
| PCT_OF_ALL_RUNS | Runs annotated with TYPE/VALUE, as a percentage of all runs |
| SAMPLE_IDS | BioSamples DB ID’s corresponding to BIOREP_ID |

# Baseline Expression Per Gene – for Tissue, Cell Type, Developmental Stage, Sex and Strain

## Format

| Item | Description |
| --- | --- |
| <http://www.ebi.ac.uk/fg/rnaseq/api> | http://www.ebi.ac.uk/fg/rnaseq/api/FORMAT/MIN_NUMBER_OF_RUNS/getExpression… |
| FORMAT | tsv or json |
| MIN_NUMBER_OF_RUNS | Reported expression is a median of expressions (TPM) across all runs corresponding to a given condition. This filter excludes conditions with less that the specified minimum number or runs. |
| ORGANISM | Species of the gene symbol provided (‘**any**’ for all species) |
| GENE_SYMBOL | Gene symbol in ORGANISM to select expression of |

## Example Calls to Retrieve Baseline Expression Per Gene

| URL |
| --- |
| <http://www.ebi.ac.uk/fg/rnaseq/api/tsv/50/getExpression/homo_sapiens/REG1B> |
| <http://www.ebi.ac.uk/fg/rnaseq/api/json/0/getExpression/oryza_sativa/BURP7> |
| [http://www.ebi.ac.uk/fg/rnaseq/api/tsv/10/getExpression/any/ALB](http://www.ebi.ac.uk/fg/rnaseq/api/tsv/50/getExpression/any/ALB) |
| <http://www.ebi.ac.uk/fg/rnaseq/api/tsv/0/getExpression/ENSG00000172023> |

## Example Calls to Retrieve All Organisms with Expression Data

| URL |
| --- |
| <http://www.ebi.ac.uk/fg/rnaseq/api/tsv/getExpressionOrganisms> |

## Returned Fields

| Field | Description |
| --- | --- |
| GENE_ID | Ensembl gene identifier |
| ORGANISM | GENE_ID’s species |
| MEDIAN EXPRESSION | Median expression value for GENE_ID, aggregated across expressions (TPM) in all sequencing runs corresponding to the reported condition (i.e. tissue, cell type, developmental stage, sex and strain - see below) |
| COEFFICIENT_OF_VARIATION | Measure of dispersion of individual runs’ expressions in around the expression mean across all runs. It is calculated as: (standard deviation) / mean. The lower its value, the more consistent the expression is across multiple runs. |
| NUM_OF_RUNS | Number of runs corresponding to the reported condition |
| ORGANISM_PART | Tissue (NA if no value available) |
| CELL_TYPE | Cell type (ditto) |
| DEVELOPMENTAL_STAGE | Developmental stage (ditto) |
| SEX | Sex (ditto) |
| STRAIN | Strain (NA if no value available or not applicable) |
| ALL_SAMPLE_ATTRIBUTES | The API link to display all sample attributes associated with runs aggregated for the reported condition, e.g. <http://www.ebi.ac.uk/fg/rnaseq/api/tsv/getSampleAttributesByCondition/1178> |
| REFERENCE_SOURCE | The source of the genome reference used in the analysis (C.f. [Ensembl](http://www.ebi.ac.uk/fg/rnaseq/api/tsv/0/getOrganisms/ensembl), [Plants](http://www.ebi.ac.uk/fg/rnaseq/api/tsv/0/getOrganisms/plants), [Fungi](http://www.ebi.ac.uk/fg/rnaseq/api/tsv/0/getOrganisms/fungi), [Metazoa](http://www.ebi.ac.uk/fg/rnaseq/api/tsv/0/getOrganisms/metazoa), [Protists](http://www.ebi.ac.uk/fg/rnaseq/api/tsv/0/getOrganisms/protists) and [WormBase ParaSite](http://www.ebi.ac.uk/fg/rnaseq/api/tsv/0/getOrganisms/wbps)) |

# Mapping Quality Statistics Across All Organisms

## Format

| Item | Description |
| --- | --- |
| URL | <http://www.ebi.ac.uk/fg/rnaseq/api/tsv/getOrganismsMappingQuality> |
| FORMAT | tsv or json |

## Returned Fields

| Field | Description |
| --- | --- |
| ORGANISM | Organism |
| MEAN_MAPPING_QUALITY | Average mapping quality across all analysed runs for the organism |
| STDDEV_MAPPING_QUALITY | Standard deviation of mapping quality across all analysed runs for the organism |

# Acknowledgements

The initial work to develop this API and perform the RNA-seq was funded by the [BBSRC](http://www.bbsrc.ac.uk/), for which we express our gratitude. We would also like to thank the [Non-vertebrate Genomics Team](http://www.ebi.ac.uk/about/people/paul-kersey) for obtaining the funding and their work on displaying the resulting CRAM files in Ensembl Plants track hubs; to the [European Nucleotide Archive Team](http://www.ebi.ac.uk/about/people/guy-cochrane) for facilitating access to the raw RNA-seq data; and to the [Samples, Phenotypes and Ontologies Team](http://www.ebi.ac.uk/about/people/helen-parkinson) for the provision of tools for retrieval of the sequencing metadata from [BioSamples](http://www.ebi.ac.uk/biosamples) database and up-to-date annotation of sequencing meta-data to [Experimental Factor Ontology](http://www.ebi.ac.uk/efo/). Finally, a big thank you is due to members of the [Gene Expression Team](http://www.ebi.ac.uk/about/people/robert-petryszak) without whom none of this would have been possible.
