# Supplementary material for: The RNASeq-er API—a gateway to systematically updated analysis of public RNA-seq data
Source: Bioinformatics. 2017 Mar 22;33(14):2218–20. doi: 10.1093/bioinformatics/btx143 (PMC5870697; doi:10.1093/bioinformatics/btx143)
Supplement: Supplementary Data [file btx143_supp.zip › btx143-suppl_data/RNASeq_API_2016_Application_Note_supplementary_Commands.docx]

**The RNASeq-er API - a gateway to systematically updated analysis of public RNA-Seq data**

Robert Petryszak^1,*^, Nuno A. Fonseca^1^, Anja Füllgrabe^1^, Laura Huerta^1^, Maria Keays^1^, Y. Amy Tang^1^, Alvis Brazma^1^

^1^ European Molecular Biology Laboratory, European Bioinformatics Institute, EMBL-EBI, Hinxton, UK

**Unix Commands used to generate results returned by the RNASeq-er API – for a single sequencing run: $run in which organism: $organism was studied:**

1. Download and install the iRAP pipeline (<http://nunofonseca.github.io/irap/>)
2. Download $organism.conf from <ftp://ftp.ebi.ac.uk/pub/databases/arrayexpress/data/atlas/rnaseq/configs>
   into $irapData/configs (where $irapData is a directory of your choosing)
3. Download into $irapData/reference/$organism directory the genome reference and the gtf file from Ensembl, Ensembl Genomes or Wormbase ParaSite as appropriate
4. For each organism of interest, please run the following command to generate appropriate indexes needed for mapping to genome reference:

   *irap_single_lib -0 -A -t $numThreads -m $memory -c $irapData/configs/$organism.conf*

where:
$numThreads – the number of threads used during indexing
$memory – the memory (in MB) required for indexing
$irapData/configs/$organism.conf – the path to the configuration file corresponding to the organism of interest

1. Run the following command to obtain the full results for a single sequencing run:
   1. Single-end run:

*irap_single_lib -A -f -1 $run.fastq.gz -c $irapData/configs/$organism.conf -s
$strand -m $memory -t $numThreads -C -i data_dir=$irapData –l $minReadLength*

- 1. Paired-end run:

*irap_single_lib -A -f -1 $run_1.fastq.gz -2 $run_2.fastq.gz -c $irapData/configs/$organism.conf -s $strand -m $memory -t $numThreads
-C -i data_dir=$irapData –l $minReadLength*

where:
$numThreads – the number of threads used during indexing
$memory – the memory (in MB) required for indexing
$irapData/configs/$organism.conf – the path to the configuration file corresponding to the organism of interest
$run.fastq.gz, $run_1.fastq.gz and $run_2.fastq.gz – paths to the single- and paired-end FASTQ files accordingly

$strand – first, second or both (to indicate if the sequencing procedure was stranded, and if so, which strand the reads should be mapped to)

$irapData – user directory that contains configuration files and genome references

$minReadLength – minimum read length considered to be valid for bulk mRNA-seq studies. We use $minReadLength=22 in our analysis.

An up-to-date information on the above commands can be found at: https://github.com/nunofonseca/irap/wiki/iRAP-single-library
